# Divergent Selection for Seed Ability to Germinate at Extreme Temperatures in Perennial Ryegrass (*Lolium perenne* L.)

**DOI:** 10.3389/fpls.2021.794488

**Published:** 2022-01-31

**Authors:** Wagdi Ghaleb, Philippe Barre, Béatrice Teulat, Lina Qadir Ahmed, Abraham J. Escobar-Gutiérrez

**Affiliations:** ^1^INRAE, URP3F, F-86600 Lusignan, France; ^2^Biotechnology Research Center (BTRC), Tripoli, Libya; ^3^Univ. Angers, Institut Agro, INRAE, IRHS, SFR QUASAV, F-49000 Angers, France

**Keywords:** divergent selection, germination, temperature, perennial ryegrass, *Lolium perenne* L., thermoinhibition

## Abstract

Various adaptive mechanisms can ensure that seedlings are established at the most favourable time and place. These mechanisms include seed dormancy i.e., incapacity to germinate in any environment without a specific environmental trigger and inhibition i.e., incapacity to germinate in an unfavourable environment (water availability, temperature: thermoinhibition and light). The objective of this research was to study in the temperate range for germination of forage and turf grass species perennial ryegrass, if the thermal requirements for germination are under genetic controlled and could be selectively bred. Two divergent selections of three cycles were realized on a natural population: one to select for the capacity to germinate at 10°C vs. the impossibility to germinate at 10°C, and one to select for the capacity to germinate at 32°C vs. the impossibility to germinate at 32°C. Seeds of all the lots obtained from the two divergent selections were then germinated at constant temperatures from 5 to 35°C to evaluate their germination ability. Concerning the positive selection, the first cycle of positive selection at 10°C was highly efficient with a very strong increase in the germination percentage. However, afterward no selection effect was observed during the next two cycles of positive selection. By contrast, the positive selection at 32°C was efficient during all cycles with a linear increase of the percentage of germination at 32°C. Concerning the negative selection, we observed only a large positive effect of the first cycle of selection at 10°C. These findings demonstrate that seed thermoinhibition at 10 and 32°C observed in a natural population of perennial ryegrass has a genetic basis and a single recessive gene seems to be involved at 10°C.

## Introduction

As the first stage of the plant life cycle, germination is particularly critical for successful establishment and growth of plants (Ooi, 2012; Nunes et al., 2016). Germination starts with water uptake by the dry seed (imbibition), continues with biochemical preparative processes and the elongation of the embryonic axis, and terminates with protrusion of the radicle out of the seed (Bewley and Black, 1994; Bewley et al., 2013). To be successful, germination has to be triggered in appropriate environmental conditions.

Seed germination ecology is a complex subject that aims to understand what controls the timing of seed germination in nature (Baskin and Baskin, 2014; Nunes et al., 2016). Various adaptive mechanisms ensure that seed germination occurs at the most advantageous time and place. These mechanisms could be classified as (i) “dormancy” that requires a special event or “trigger” before the embryo can resume growth e.g., stratification by cold temperatures and (ii) “inhibition” that requires just favourable environmental conditions for the embryo to grow (Hills and van Staden, 2003; Batlla and Benech-Arnold, 2015). Consequently, for some dormant seeds, special environmental conditions must be met to release this dormancy and then additional conditions must be met to allow germination of the seeds (Baskin and Baskin, 1988; Simpson, 1990; Bewley, 1997; Bewley et al., 2013). Important environmental factors influencing the inhibition of seed germination are water availability, temperature and light. In particular, the action of temperature on seed germination is related to enzyme activity and membrane stability and permeability affecting respiratory metabolism (Baskin and Baskin, 2014; Nunes et al., 2016).

The response of germination to temperature in different species has been largely studied (Bewley and Black, 1994; Brunel et al., 2009; Durr et al., 2015; Tribouillois et al., 2016; Ghaleb, 2019; Ghaleb et al., 2021). Major differences among species depend on the climatic conditions where the species originate. Furthermore, cultivated species germinate faster and under a wider range of temperatures than wild species, likely as a result of human selection (Durr et al., 2015; Ghaleb et al., 2021). The same tendency of various response of germination to temperature has been observed among populations of the same species (Butler et al., 2017; Ahmed and Escobar-Gutiérrez, 2022). In perennial ryegrass (*Lolium perenne* L.), it has been shown that the capacity of germination (after dormancy release) at different temperatures varies dramatically between natural populations of various geographical origins (Ghaleb, 2019). The variability within populations is also noticeable. For example, within the 3038 population, 95% of the seeds (different genotypes) do not germinate at a sub-optimal temperature (e.g., 5°C) whereas the remaining 5% germinate. Varieties of perennial ryegrass tend to germinate at a high rate at almost all temperatures (Ahmed et al., 2014, “accession identifier is ACVF60016”; Ghaleb, 2019, “accession identifier is 553”). These observations at different levels of genetic variability demonstrate that the response of germination to temperature is at least partly genetically controlled.

The heritability and the genetic determinism of the response of germination to temperature is still not completely understood (Baskin and Baskin, 2014; Yan and Chen, 2020; Ghaleb et al., 2021). It has been shown that it is possible to select for germination at a given temperature, for example in maize at low temperature (9.5°C) (Landi et al., 1992; Frascaroli and Landi, 2013). Several works, using mapping populations (e.g., Hayashi et al., 2008; Argyris et al., 2011; Dias et al., 2011; Jiang et al., 2017) and natural populations association studies (e.g., Chopra et al., 2017), have reported QTLs for the ability to germinate at specific temperatures. These results are encouraging for breeding for the ability or not to germinate at specific temperature.

Perennial ryegrass is the most sown grass forage species in temperate climates (Wilkins, 1991; Jones et al., 2002; Zhang et al., 2006). Natural populations are distributed in a wide range of environments across Europe and the Mediterranean regions (Wilkins, 1991; Blackmore et al., 2016; Blanco-Pastor et al., 2019). Consequently, vast natural genetic resources exist in this species (Blanco-Pastor et al., 2019) that can be used to produce genetic material with new traits adapted to new climatic constraints such as the inhibition of seed germination at extreme temperatures and meeting the needs of agriculture (Keep et al., 2020). Perennial ryegrass is an obligate outcrossing species for which varieties are synthetics (intercrosses of several selected individuals). As a consequence, genetic diversity exists in both natural populations and varieties (e.g., Kubik et al., 2001; Bolaric et al., 2005).

The objective of this research was to study if the ability of perennial ryegrass to germinate or not at a given temperature was genetically controlled and could be selected. For this purpose, two divergent selections (one at 10°C and one at 32°C) were realized on the 3,038 population of perennial ryegrass showing contrasting germination ability at different temperatures. Some seeds (genotypes) have the ability to germinate at 10°C or at 32°C whereas others do not, thus, revealing the variability within this population for this trait. Almost all seeds have the ability to germinate at 25°C after incubation at sub/supra-optimal temperatures, indicating that the absence of germination at low or high temperatures should be interpreted as an inhibition of germination and not as a dormancy, which has been released by a cold treatment (5°C during 7 days) (ISTA, 2015).

### Plant Material

**FIGURE 1:**
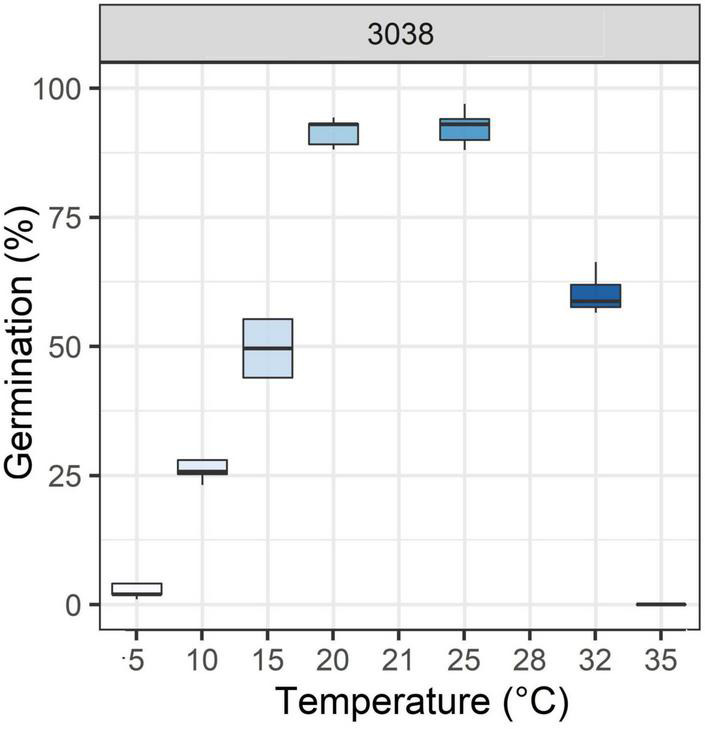
Boxplots of germination percentage in response to different temperatures of the 3038 wild population of perennial ryegrass (Ahmed et al., 2014; Ghaleb, 2019).

In this study, a wild population of perennial ryegrass (*Lolium perenne* L.) (accession 3038) was used. Seeds were obtained from INRAE’s Centre de Ressources Biologiques des Espèces Fourragères (CRB) at Lusignan, France. The coordinates of the site of collection are 49°15′29.98″N, 4°01′54.11″E. This population was chosen for its variable germination capacities in relation to temperature (Figure 1). Seeds were produced in 1991 and conserved at 5°C and 30% relative humidity until they were used.

### Divergent Selections

Regarding the response of germination capacity to temperature on the 3038 population, two divergent selections were performed: one to select for the capacity to germinate at 10°C vs. the impossibility to germinate at 10°C (inhibition of seed germination at 10°C), and one to select for the capacity to germinate at 32°C vs. the impossibility to germinate at 32°C (inhibition of seed germination at 32°C). The germination protocol is explained in the following part (Germination experiment).

**FIGURE 2:**
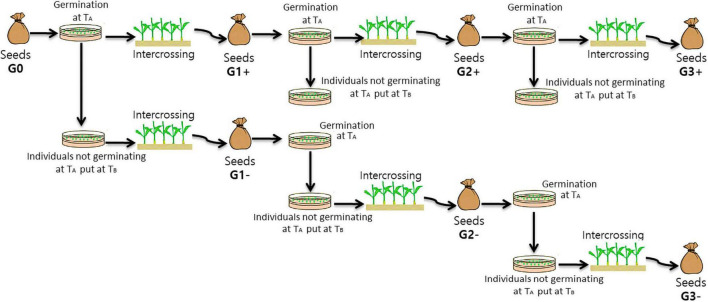
Diagram illustrating the steps of the divergent selections for the capacity to germinate or not at an unfavourable temperature. From G0 seeds (initial generation/generation 0) to G3 seeds (third generation of selection) (T_A_ = Unfavourable temperature = 10°C or 32°C and T_B_ = Optimum temperature = 25°C).

Three cycles of divergent selection were performed (Figure 2):

- In the first cycle about 1000 seeds from the initial population were placed at an sub/supra-optimal temperature T_A_ (10 or 32°C) for germination and seedlings obtained from germinated seeds were selected. These grown-up plants were then intercrossed to form the positive selection (PS) of the next generation (G1+ obtained in 2015). In parallel, seeds that did not germinate at T_A_ were placed at an optimal germination temperature T_B_ (25°C), and seedlings obtained from germinated seeds were selected. These plants were then intercrossed to form the negative selection (NS) of the next generation (G1− obtained in 2015).
- In cycles two and three, 1000 seeds of PS were placed at T_A_ and seedlings obtained from germinated seeds were selected. These grown-up plants were then intercrossed to form PS of the next generation (G2+ obtained in 2016 and G3+ obtained in 2017). The other seeds were placed at T_B_ to evaluate the total germination capacity. In the other hand about 1000 seeds from NS were placed at T_A_ and the seeds that did not germinate at T_A_ were placed at an optimal germination temperature T_B_ and seedlings that have germinated were selected. Grown-up plants were then intercrossed to form NS of the next generation (G2− obtained in 2016, no G3− were obtained due to the delay necessary to select the plants before intercrossing). Numbers of individuals intercrossed at each cycle of the divergent selection are indicated in Table 1.

**TABLE 1:** Number of individuals (from 1000 seeds) intercrossed at each cycle of the divergent selections.

	Cycle one	Cycle two	Cycle three
**Selection at 32°C**			
Positive selection	115	159	215
Negative selection	39	278	205
**Selection at 10°C**			
Positive selection	130	372	260
Negative selection	224	372	267

### Germination Experiment

Seeds of all the lots obtained from the two divergent selections were germinated at constant temperatures from 5 to 35°C to evaluate their germination ability (in this assessment the initial lot of 1991 was replaced by a new lot obtained in 2014, for excluding the effect of seed age on seed germination). These large ranges of temperatures were conducted to evaluate the effect of divergent selection at one temperature on the ability to germinate at different temperatures. The germination test was performed between July and December 2019. Seeds of G1 were obtained in July 2016, the ones of G2 were obtained in July 2017, and the ones of G3 were obtained in July 2018.

For each temperature, every seed-lot was represented by five replicates of 100 seeds, which were arranged in a completely randomized design. Seeds were placed in 90 mm diameter Petri dishes containing a sheet of autoclave-sterilized Whatman paper (ref. 3645 Whatman, France) humidified with 5 ml of deionized and autoclave-sterilized water. Seeds were cold stratified in the dark for 7 days at 5°C and 30% relative humidity (0.61 kPa of Vapour-pressure deficit, VPD) in order to release any residual seed dormancy. After cold stratification, seeds were germinated in the dark at constant temperatures of 5, 10, 15, 20, 25, 32, and 35°C in 1.5 m^3^ useful-volume growth chambers (Froids et mesures, France). Temperature measurements from five to six thermocouples placed at different positions within each growth chamber, were logged every 20 s. Each growth chamber was regulated so that its temperature (average measurement from the thermocouples) did not exceed ± 0.2°C of the target temperature. The frequency of sampling for germination depended on temperature and the number of non-germinated seed within each Petri dish. Seeds were considered as germinated when either the radicle or the coleoptile had protrusion out of the seed and was at least 2 mm long. At each counting, germinated seeds were counted and removed from the Petri dishes. Deionized and autoclave-sterilized water was added as required to ensure moisture was non-limiting for germination.

### Statistical Analysis

The data of germination percentages were analysed by two-way analysis of variance (ANOVA) and a *post hoc* Tukey’s test with significance level set at *P* < 0.05 was carried out to examine the effects of generation (i.e., cycle of selection), temperature and interaction of both factors on the divergent selection. The germination percentage of a Petri dish containing 100 seeds was considered as the basic datum. In addition, a model with population as random effect was performed to estimate genetic and residual variances at 10°C for the divergent selection at 10°C and at 32°C for the divergent selection at 32°C. The variances were used to calculate heritability as genetic variance divided by total variance. All calculations and statistical tests were carried out using R software (version 4.0.3; R Core Team, 2020).

## Results

**FIGURE 3:**
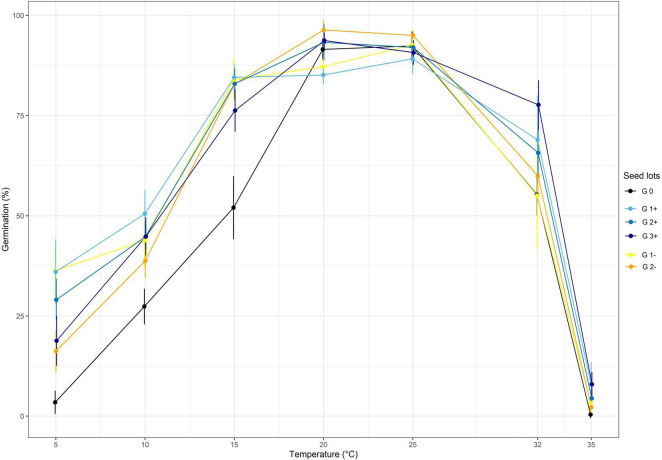
The effect of divergent selection at 32°C on the seed germination percentage of all the populations obtained from positive selection (G1+, G2+, G3+) and negative selection (G1-, G2-). G0 corresponds to the initial population.

**FIGURE 4:**
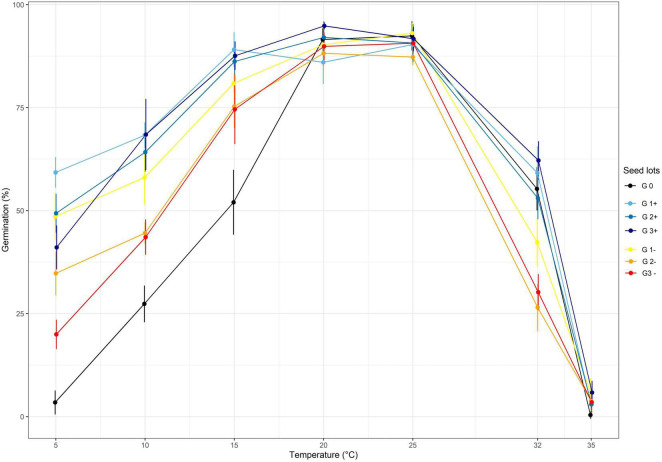
The effect of divergent selection at 10°C on the seed germination percentage of all the populations obtained from positive selection (G1+, G2+, G3+) and negative selection (G1-, G2-). G0 corresponds to the initial population.

Two divergent selections were performed at two targeted temperatures: T_A_ = 10°C and T_A_ = 32°C and using the same optimum temperature T_B_ = 25°C. The effect of the divergent selections at each generation are shown in Figures 3, 4 for the selection at T_A_ = 32°C and the selection at T_A_ = 10°C, respectively.

In order to better assess the effect of selection, the germination capacity of the different populations resulting from the selection and the initial population, was evaluated in the range of temperatures from 5 to 35°C. The heritability were 89.7 and 87.7% for the divergent selection at 10 and 32°C, respectively.

Regarding the divergent selection at 32°C, significant effects of generations, temperature and the interaction of both factors on the germination percentage were established using a two-way ANOVA (Table 2 and Figure 3). A significant increase of germination percentage at 32°C was observed during the three cycle of PS going from 55.3% for G0 to 77.7% for G3+, showing a clear effect of selection (Supplementary Table 1). By contrast, the effect of the NS was not significant at 32°C. The first cycle of PS and NS at 32°C significantly increased the percentage of germination at temperatures below 20°C.

**TABLE 2:** Two-way ANOVA of the germination percentage used to evaluate the effect of divergent selection at 32 and 10°C.

Source	DF	Sum of squares	Mean squares	*F* value
**Divergent selection at 32°C**				
Generation	5	5,542	1,108	33.20***
Temperature	6	210,863	35,144	1,052.74***
Generation × Temperature	30	8,811	294	8.71***
**Divergent selection at 10°C**				
Generation	6	14,842	2,474	121.1***
Temperature	6	215,894	35,982	1,761***
Generation × Temperature	36	20,894	580	28.4***

***Significant at P < 0.001.

Regarding the divergent selection at 10°C, significant effects of generations, temperature and the interaction of both factors on the germination percentage were established using a two-way ANOVA (Table 2 and Figure 4). An important significant increase of germination percentage at 10°C was observed during the first cycle of PS going from 27.4 to 68.3%, showing a clear effect of selection (Supplementary Table 2). Afterward, no significant increase of germination percentage at 10°C was observed during the two following cycles of selection. Surprisingly, we observed an increase of germination percentage at 10°C after the first cycle of NS. Then, as expected, the percentage of germination at 10°C decreased after the second cycle of NS but no significant effect of selection was observed after the third cycle of NS. The first cycle of PS and NS at 10°C significantly increased the percentage of germination at low temperatures: 5, 10 and 15°C. No effect of selection was observed at 20 and 25°C for which the percentage of germination was high (>85%) for all seed lots showing their good quality.

## Discussion

This experimental study showed clearly that the ability to germinate at specific unfavourable temperatures could be selectively bred within a natural population of perennial ryegrass. This implies the existence of a genetic variability for the temperature at which the inhibition of seed germination is released. This variability could be essential for the adaptation of the population to inter-annual climatic variation e.g., some years it could be beneficial to germinate at 10°C even if in the majority of the years it is detrimental due to late frost. Moreover, in a longer term, this variability could allow adaptation to climate change. Few examples of within species genetic variability of the ability to germinate at different temperatures have been reported (Hampton et al., 1987; Palazzo and Brar, 1997; Lonati et al., 2009; Zhang et al., 2013; Nori et al., 2014; Butler et al., 2017; Ahmed et al., 2019; Ghaleb, 2019; Ahmed and Escobar-Gutiérrez, 2022; Ghaleb et al., 2021). In some cases, the genetic nature of this variability has been demonstrated by divergent selections like in alfalfa and maize for the ability to germinate at low temperatures (McConnell and Gardner, 1979; Landi et al., 1992; Klos and Brummer, 2000; Frascaroli and Landi, 2013).

Our results showed that the genetic determinism of the regulation of the release of seed germination inhibition involved at least one “major” gene at 10°C and several “minor” genes at 32°C. The first cycle of PS at 10°C was highly efficient with a very strong increase in the germination percentage (from 27.4 to 68.3%). However, afterward no selection effect was observed during the following two cycles of positive selection. These results could be explained by the presence of a dominant gene (D) inhibiting the germination of seeds at low temperatures. The allele frequency of the recessive allele (d) would be about 52% in the G0 population (square root of 27.4%: the percentage of individuals without any thermoinhibition of seed germination). The absence of response after the first cycle of selection could be explained by the fixation of the recessive (thermoinhibition of seed germination) allele. This “major” gene would explain only a part of the variability since 22–26% of the genotypes from G2+ and G3+ showed a thermoinhibition of seed germination despite the fact that they should be fixed at this “major” gene (dd). “Minor” genes and the environment could be involved in order to explain the thermoinhibition of seed germination of these genotypes. A “major” recessive gene could explain the fact that the varieties have generally lost this thermoinhibition of seed germination (only one generation of selection is needed). By contrast, the PS at 32°C showed a linear increase of the percentage of germination at 32°C (*R*^2^ = 0.79) despite a relatively stronger effect of the first cycle of selection. This result could be explained by the progressive accumulation of no thermoinhibition alleles at several loci.

The control of the temperature requirements for germination (i.e., thermoinhibition) at low temperatures by a “major” gene has been observed in different species. For example, the ability to germinate at 10°C of *Lycopersicon esculentum* seeds is controlled by a single recessive gene (Baskin and Baskin, 2014). In studies on the germination requirements of *Avena fatua*, A. *ludoviciana* and their hybrids, hypotheses were made regarding the number of genes involved in the temperature requirement for dormancy breaking. Failure of A. *fatua* to germinate at 5°C is probably controlled by three recessive loci, and failure of A *ludoviciana* to germinate at 18°C seems to be controlled by a single recessive gene (Whittington et al., 1970). In other studies, a major QTL has been identified for thermotolerance in lettuce and cucumber seeds (O’Brien, 2007; Argyris et al., 2008; Yoong et al., 2016; Yagcioglu et al., 2019). In maize, Hu et al. (2016) identified three QTL for optimum temperature germination rate and six QTL for low temperature germination rate.

The results on the NS were surprising. No consequent effect was observed except a large positive effect of the first cycle of selection at 10°C i.e., the percentage of germination at 10°C went from 27.4 to 58% whereas we selected for seeds unable to germinate at 10°C (Figure 4). This might be due to the fact that we selected the first seeds which germinated after their transfer from 10 to 25°C. Some of these individuals could be lagged not inhibited individuals which should have germinated at 10°C but for some reason were delayed. These individuals would carry the *dd* alleles.

In conclusion, we have demonstrated that thermoinhibition of seed germination at 10 and 32°C in a natural population of perennial ryegrass has a genetic basis and a single recessive gene seems to be involved at 10°C. It would be interesting to confirm this result in other natural populations showing a similar thermoinhibition at low temperatures. In addition, human selection has been efficient to suppress the thermoinhibition of seed germination so that seeds germinate in a wide range of temperature conditions. Nevertheless, it should be possible to select for the thermoinhibition of seed germination to avoid germination at unfavourable temperatures. For example, it could be interesting to select for varieties unable to germinate at high temperature so that these varieties could be sown at the end of the summer when the temperatures are still unfavourable for germination and that seeds germinate only when the temperatures decrease in autumn. At 10°C it would be helpful to develop a molecular marker in order to differentiate rapidly the Dd and DD genotypes showing both a phenotype with thermoinhibition of seed germination.

## Data Availability Statement

The datasets presented in this study can be found in online repositories. The names of the repository/repositories and accession number(s) can be found below: https://data.inrae.fr/dataset.xhtml?persistentId=doi:10.15454/MO7RP9, 10.15454/MO7RP9.

## Author Contributions

AE-G and PB conceived the study. AE-G, PB, and WG designed the experiment. WG collected the data with contributions of PB, AE-G, and LQA. WG and PB carried out the data analysis. PB, AE-G, and LQA contributed to write the manuscript. All authors contributed to the article and approved the submitted version.

## Conflict of Interest

The authors declare that the research was conducted in the absence of any commercial or financial relationships that could be construed as a potential conflict of interest.

## Publisher’s Note

All claims expressed in this article are solely those of the authors and do not necessarily represent those of their affiliated organizations, or those of the publisher, the editors and the reviewers. Any product that may be evaluated in this article, or claim that may be made by its manufacturer, is not guaranteed or endorsed by the publisher.
