# Supplementary material for: Divergent Selection for Seed Ability to Germinate at Extreme Temperatures in Perennial Ryegrass (Lolium perenne L.)
Source: Front Plant Sci. 2022 Jan 31;12:794488. doi: 10.3389/fpls.2021.794488 (PMC8841656; doi:10.3389/fpls.2021.794488)
Supplement: Supplementary file 1 [file Table_1.DOCX]

Supplementary Table 1: Comparison the effect of divergent selection on the germination percentage at 32 °C of between the populations obtained from PS (G1+, G2+,G3+) and NS (G1-,G2-,G3-) and initial lot (G0).

| **Temperature (°C)** | **Generations** | | | | | | | | | | | | |
| --- | --- | --- | --- | --- | --- | --- | --- | --- | --- | --- | --- | --- | --- |
|  | **G0** | **G1+** | | | **G2+** | | **G3+** | | **G1-** | | **G2-** | | **G3-** |
| **5** | 03.4 d | | 36.0 a | 29.0 ab | | 18.8 bc | | 36.2 a | | 16.2 c | | Na | |
| **10** | 27.4 c | | 50.5 a | 44.8 ab | | 44.8 ab | | 43.8 ab | | 38.8 b | | Na | |
| **15** | 52.0 b | | 84.5 a | 82.9 a | | 76.3 a | | 83.9 a | | 82.9 a | | Na | |
| **20** | 91.5 ab | | 85.1 c | 93.3 a | | 93.7 a | | 87.2 bc | | 96.4 a | | Na | |
| **25** | 92.4 ab | | 89.2 b | 92.0 ab | | 90.7 ab | | 92.6 ab | | 95.0 a | | Na | |
| **32** | 55.3 c | | 69.0 ab | 65.7 bc | | 77.7 a | | 55.0 c | | 59.9 bc | | Na | |
| **35** | 00.4 b | | 08.0 a | 04.4 ab | | 07.9 a | | 03.3 ab | | 02.2 ab | | Na | |

Values followed by different letters indicate significant differences between generations at *P*<0.05

Supplementary Table 2: Comparison the effect of divergent selection on the germination percentage at 10 °C of between the populations obtained from PS (G1+. G2+.G3+) and NS (G1-.G2-.G3-) and initial lot (G0) of the ACVF 60016 population.

| **Temperature (°C)** | **Generations** | | | | | | |
| --- | --- | --- | --- | --- | --- | --- | --- |
|  | **G0** | **G1+** | **G2+** | **G3+** | **G1-** | **G2-** | **G3-** |
| **5** | 03.4 e | 59.3 a | 49.4 b | 41.1 bc | 48.6 b | 34.8 c | 20.0 d |
| **10** | 27.4 c | 68.3 a | 64.2 a | 68.5 a | 58.0 a | 44.6 b | 43.6 b |
| **15** | 52.0 c | 89.1 a | 86.2 a | 87.6 a | 80.9 ab | 75.3 b | 74.6 b |
| **20** | 91.5 ab | 86. b | 92.0 ab | 94.8 a | 90.2 ab | 88.2 b | 89.9 ab |
| **25** | 92.4 a | 90.3 a | 90.7 a | 91.7 a | 93.1 a | 87.3 a | 90.6 a |
| **32** | 55.8 ab | 59.2 ab | 53.0 b | 62.2 a | 42.3 c | 26.5 d | 30.2 d |
| **35** | 0.4 b | 03.0 ab | 3.0 ab | 05.8 ab | 05.8 a | 03.8 ab | 03.5 a |

Values followed by different letters indicate significant differences between generations at *P*<0.05
